# Stability of Breathers for a Periodic Klein–Gordon Equation

**DOI:** 10.3390/e26090756

**Published:** 2024-09-04

**Authors:** Martina Chirilus-Bruckner, Jesús Cuevas-Maraver, Panayotis G. Kevrekidis

**Affiliations:** 1 Mathematisch Instituut, Universiteit Leiden, P.O. Box 9512, 2300 RA Leiden, The Netherlands; m.chirilus-bruckner@math.leidenuniv.nl; 2 Grupo de Física No Lineal, Departamento de Física Aplicada I, Universidad de Sevilla, Escuela Politécnica Superior, C/Virgen de África, 7, 41011 Sevilla, Spain; 3 Instituto de Matemáticas de la Universidad de Sevilla (IMUS), Edificio Celestino Mutis. Avda. Reina Mercedes s/n, 41012 Sevilla, Spain; 4 Department of Mathematics and Statistics, University of Massachusetts Amherst, Amherst, MA 01003, USA; kevrekid@umass.edu

**Keywords:** nonlinear Klein-Gordon PDE, spectral stability, breathers, heterogeneous media, center manifold reduction

## Abstract

The existence of breather-type solutions, i.e., solutions that are periodic in time and exponentially localized in space, is a very unusual feature for continuum, nonlinear wave-type equations. Following an earlier work establishing a theorem for the existence of such structures, we bring to bear a combination of analysis-inspired numerical tools that permit the construction of such waveforms to a desired numerical accuracy. In addition, this enables us to explore their numerical stability. Our computations show that for the spatially heterogeneous form of the $\phi^{4}$ model considered herein, the breather solutions are generically unstable. Their instability seems to generically favor the motion of the relevant structures. We expect that these results may inspire further studies towards the identification of stable continuous breathers in spatially heterogeneous, continuum nonlinear wave equation models.

## 1. Introduction

The sine-Gordon model is a quintessential example of a dispersive partial differential equation model within nonlinear science that has been explored in numerous reviews [1], as well as books [2,3,4]. One of the very well-known and exciting features of this integrable (via inverse scattering [5]) equation is the existence of exact breather solutions. These are temporally periodic, exponentially spatially localized waveforms that are known in an explicit analytical form in this model.

The presence of such breathers has been recognized in spatially discrete models as a rather generic feature, ever since the work of Sievers-Takeno [6], Page [7] and many others. Indeed, not only has this work spearheaded applications in areas ranging from optical waveguide arrays to superconducting Josephson junctions, atomic condensates, DNA and beyond, but it has also inspired numerous reviews summarizing the pertinent progress (see, e.g., [8,9]).

On the other hand, in a classic paper from 30 years ago, Birnir, McKean and Weinstein showed a quite remarkable result [10], namely that only perturbations of the integrable sine-Gordon (sG) model of the forms sin(u), ucos(u) and 1+3cos(u)−4cos(u/2)+4cos(u)log(cos(u/4)) can give rise to breathing waveforms. The first two of these stem from rescalings of the standard sG breather, while the third one is believed to be impossible. This suggests that breathers are rather non-generic in continuous problems. Indeed, in a sense, this is intuitively understandable. On the one hand, a breather has an intrinsic frequency associated with it; on the other hand, the background state on which it lies has a continuous spectrum of plane-wave excitations. Generically, the intrinsic breather frequency or, most typically, its (nonlinearly induced) harmonics find themselves in resonance with the continuous spectrum, opening a channel of “radiative decay” for the breather. That is the principal reason why in a “classic” model such as the $\phi^{4}$ model, one of the celebrated results concerns the non-existence of breathers in the model (at least in a truly localized form) on account of such a resonance [11]. That being said, in the sG case, the “magic” of integrability precludes the activation of such resonances and leads to the persistence of the exact breather waveform.

More recently, these classic findings have prompted a renewed interest in seeking to identify continuum (but now heterogeneous) models in which one can rigorously establish the existence of such breather waveforms. This was initiated in a study of one of the present authors [12] and was continued by other groups via different types of (variational) methods [13], yet the fundamental principle is clear, namely to construct a heterogeneous problem such that its band structure can be identified and the frequency of the breather and its potential harmonics are non-resonant with the continuous spectral bands.

It is this vein of research that we bring to bear herein by complementing it with detailed numerical studies. Upon selecting an example that is promising for the avoidance of relevant resonances, we find the relevant breather waveform numerically. We then verify that, per the theoretical prediction, the multiples of the relevant frequency do not collide with the spectral bands. We subsequently perform spectral stability analysis of the relevant breather waveform and also delve into continuations over different waveforms among the breather (e.g., frequency) and model (e.g., the periodic potential) parameters. Interestingly, we find that in the setting considered herein, the breather waveform is spectrally unstable. Nevertheless, when exploring the dynamical evolution of the respective structures, we find that, typically, the result of instability is not the disintegration of the breather but its mobility. This is also, to some degree, surprising, given that in media where the spatial periodicity is reflected in their “discreteness”, it is well-known that so-called Peierls–Nabarro barriers hinder breather mobility [8,9]. Once again, we make the point, however, that the principal contribution herein concerns being able to bring to bear the relevant theoretical notions through the detailed computation of breather solutions of such heterogeneous settings up to a prescribed accuracy. Once this is achieved, nearly for free, we explore and characterize the breather stability (through the eigenvalues of the monodromy matrix) and its continuation as a function of the system parameters.

Our presentation of these results is structured as follows: In Section 2, we lay out the general setup of the model and also summarize the main theoretical findings. In Section 3, we present our numerical computations for the breathers, their spectral stability, their parametric continuations and their nonlinear dynamics. Finally, in Section 4, we summarize our findings and present our conclusions, as well as some directions for future studies.

## 2. Mathematical Setup and Main Theoretical Result

**Figure 1:**
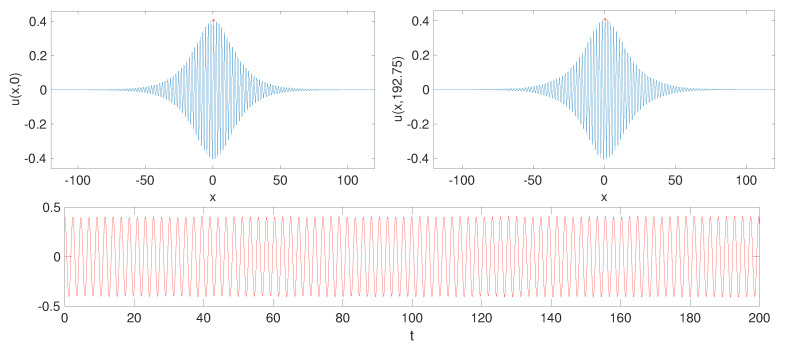
Breather spatial profiles in the upper-left and -right panels are at different times ($t=0,\;t\approx 25\cdot 2\pi /\omega_{*}$). The lower panel shows the time evolution of the center value (red dot). Coefficients are chosen to be a *resonance-free triplet* $s_{step},\;q_{step},\;\omega_{*}$ as in Definition 2, with p=0.43,ε=0.15. The initial condition is based on Proposition 1 (numerical simulation with pdepe from Matlab, R 2020a).

In line with some of the abovementioned works and the classical results in sG and $\phi^{4}$ mentioned in the Introduction, in the present section, we consider a Klein–Gordon-type equation given by

(1) $$\begin{array}{c} s\left(x\right)\partial_{t}^{2}u=\partial_{x}^{2}u-q\left(x\right)u+\rho u^{3} \end{array}$$

for x,t,u=u(x,t)∈R with spatially 1-periodic coefficients (s,q) and some constant (ρ∈R). The physical situation that we envision here is one where the effective mass of the system and its linear restoring force are modulated in a spatially dependent/heterogeneous way so as to construct the types of patterns that we see below. In this context, it is important to also highlight that the study of such nonlinear Klein–Gordon equations with spatially inhomogeneous coefficients has been a topic of considerable interest recently, especially in the context of the sine-Gordon equation [14,15,16] and its variants [17] (such as the sinh-Gordon equation), where explicit techniques can be used to construct solitary waves for spatio-temporally variable nonlinearity coefficients. The focus of the present study is on the spectral stability of breathers (see Figure 1).

**Definition** **1** (Breather solutions)**.** *We call a solution (u) of *(1)* a breather (solution) if there exist $\omega_{*}>0,\;\beta >0$ such that the following holds:*

*(i)*  *For all x,t∈R, we have $u\left(x,t\right)=u\left(x,t+\frac{2\pi }{\omega_{*}}\right)$ (“periodicity in time");*
*(ii)*  *For all t∈R, we have $\lim_{|x|\to \infty }u\left(x,t\right)e^{-\beta |x|}=0$ ((exponential) “localization in space").*

The existence of breathers for (1) was demonstrated in [12] for a specific choice of step-function coefficients (s,q) (as described in Definition 2 for the special case of $p=\frac{6}{13}$). This result seemed rather exceptional, given the “rigidity of breathers” discussed in the Introduction (in addition to [10], see also [18,19]). In fact, the construction of breathers for the case of periodic coefficients uses the tailoring of a spectral picture that would be impossible in the constant-coefficient case.

We now build up towards an extended existence result for breathers in the $\phi^{4}$ model (1), which is based on the approach proposed in [12]. The extension manifests itself in determining a whole family of step functions (see Definition 2) for which the core mechanism of the breather construction in [12], namely the tailoring of the band structure, is possible. The corresponding existence result for breathers is stated in Proposition 1. Instead of demonstrating its proof, we provide a detailed exposition of the design of the band structure.

### 2.1. Designing the Band Structure to Support Breather Solutions

Let us now turn our attention to the linear Klein–Gordon equation with periodic coefficients, where

(2) $$\begin{array}{c} s\left(x\right)\partial_{t}^{2}u=\partial_{x}^{2}u+q\left(x\right)u\;, \end{array}$$

for u=u(x,t). The related eigenvalue problem, i.e.,

(3) $$\begin{array}{c} -\omega^{2}s\left(x\right)v=\partial_{x}^{2}v+q\left(x\right)v\;, \end{array}$$

can be obtained via the ansatz

$$u\left(x,t\right)=e^{i\omega t}v\left(x\right)\;,\;\omega \in R\;.$$

A value iω∈iR belongs to the spectrum if and only if there exists bounded solutions (*v*) of (3)—in other words, when the corresponding Floquet exponents are purely imaginary (see also (4) below). It is known from Floquet–Bloch theory that the spectrum consists of a countable infinity of closed intervals, so it is “banded”, and that the corresponding solutions, the so-called Bloch waves, have the following form:

$$\begin{array}{c} u\left(x,t\right)=e^{i\omega_{n}\left(l\right)t}\;e^{ilx}\;\psi_{n}\left(x;l\right)\;,\;n\in N\;,l\in \left(-\pi ,\pi \right]\;, \end{array}$$

with $\psi_{n}\left(x;l\right)$ representing a periodic function in *x* with the same period as the underlying coefficients and ${\left(\omega_{n}\left(l\right)\right)}_{n\in N}$ corresponding to a collection of spectral bands fulfilling

$$\omega_{n}\left(l\right)\leq \omega_{n+1}\left(l\right),l\in \left(\pi ,\pi \right]\;.$$

Using Floquet theory, one can determine the band structure $\left(l,\omega_{n}\left(l\right)\right)$ via

(4) $$\begin{array}{c} e^{\pm il}=\frac{1}{2}\left(D\left(\omega \right)\pm \sqrt{D{\left(\omega \right)}^{2}-4}\right)\;, \end{array}$$

so ±il are the Floquet exponents and

(5) $$\begin{array}{c} D\left(\omega \right)=\mathrm{trace}(\Phi_{\omega }\left(x\right){|}_{x=1}) \end{array}$$

is the Floquet discriminant with

(6) $$\begin{array}{c} \frac{d}{dx}\Phi_{\omega }\left(x\right)=\left(\begin{array}{cc} 0 & 1\\ -(q\left(x\right)+s\left(x\right)\omega^{2}) & 0 \end{array}\right)\Phi_{\omega }\left(x\right)\;,\;\Phi_{\omega }\left(0\right)=Id\;. \end{array}$$

**Figure 2:**
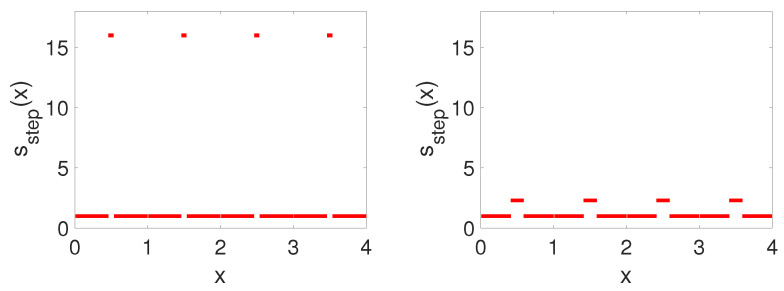
Coefficients $s_{step}$ as defined in (7) for different values of *p* (left: p=6/13≈0.4615, right: p=0.41). The closer *p* is to 1/2, the steeper and thinner the region unequal 1 is. In the limit of p→3/8, the step flattens and widens approaching the function identical 1. The steeper the step, the wider the band gaps. Numerically, it is more convenient to smooth out the steps using scaled versions of tanh(x).

In particular, any ω with |D(ω)|>2 cannot fulfill (5) for l∈R, so it must necessarily fall into a spectral gap. This connection between band structure and fundamental systems explains why it is either technically involved or impossible to obtain an explicit expression for the spectral bands of a fundamental system. The bottleneck is an explicit, workable expression for the fundamental system (6). Here, we focus on the special case of step function potentials of $s=s_{\mathrm{step}}$ as in (7) (see Figure 2).

**Lemma** **1** (Exact band structure)**.** *Consider *(2)* with q(x)=0 and s,ω, defined as follows:*

*1.*  *For p∈(3/8,1/2), let s(x+1)=s(x),x∈R and*
(7) $$\begin{array}{cc} s(x) & =s_{step}\left(x\right):=1+C\chi_{\left[p,1-p\right)}\left(x\right)\;,\;x\in \left[0,1\right)\;,\sqrt{1+C}=\frac{2p}{3(1-2p)}\\ \chi_{\left[p,1-p\right)}\left(x\right) & =\left\{\begin{array}{cc} 1, & \mathrm{if}\;p\leq x\leq 1-p\\ 0, & \mathrm{otherwise} \end{array}\right.\;. \end{array}$$
*2.*  *Let*
(8) $$\begin{array}{c} \omega =\omega_{*}\left(s\right)=\frac{\pi }{\int_{0}^{1}\sqrt{s_{step}\left(\tau \right)}\;d\tau }=\frac{3\pi }{8p}\;\left(“\mathrm{Bragg}\;\mathrm{frequency}”\right). \end{array}$$

*Then, it holds true that*

(9) $$\begin{array}{c} D\left(\omega \right):=-\frac{1}{12p(2p-1)}\left({\left(3-4p\right)}^{2}\cos\left[\frac{8}{3}p\sqrt{\omega^{2}}\right]-{\left(3-8p\right)}^{2}\cos\left[\frac{4}{3}p\sqrt{\omega^{2}}\right]\right)\;, \end{array}$$

*such that the band structure $\left(l,\omega_{n}\left(l\right)\right)$ can be obtained explicitly using *(4)*. Furthermore, we have $D\left(m\omega_{*};s\right)<-2,\;m\in N_{odd}$, so $\omega =m\omega_{*},m\in N_{odd},$ cannot fulfill the band–structure relation *(4)*, meaning that any odd multiple of the Bragg frequency is located in an odd spectral gap.*

**Proof.**  Observe that

$$\begin{array}{c} Y^{\prime }\left(x\right)=\left(\begin{array}{cc} 0 & 1\\ -\lambda r & 0 \end{array}\right)Y\left(x\right)\;, \end{array}$$

has the following fundamental matrix

$$\begin{array}{c} \Phi \left(x\right)=\left(\begin{array}{cc} \cos(\sqrt{\lambda r}\;x) & \frac{1}{\sqrt{\lambda r}}\sin\left(\sqrt{\lambda r}\;x\right)\\ -\sqrt{\lambda r}\sin\left(\sqrt{\lambda r}\;x\right) & \cos(\sqrt{\lambda r}\;x) \end{array}\right)\;. \end{array}$$

Hence, choosing $s=s_{\mathrm{step}}$ to be a step-function as in (7),

$$\begin{array}{c} D\left(\lambda ;s_{\mathrm{step}}\right)=\mathrm{trace}\left[\Phi (p)\Phi (1-2p)\Phi (p)\right]\;, \end{array}$$

and a lengthy computation yields

$$\begin{array}{cc} D(\lambda ;s_{\mathrm{step}}) & =\frac{1}{2}\left(2+\frac{2+C}{\sqrt{1+C}}\right)\cos\left[u+v\right]-\frac{1}{2}\left(-2+\frac{2+C}{\sqrt{1+C}}\right)\cos\left[u-v\right]\;, \end{array}$$

with

$$\begin{array}{c} u\pm v=\sqrt{\lambda }\left(2p\pm \sqrt{1+C}\left(1-2p\right)\right)\;. \end{array}$$

Furthermore, using the Bragg-frequency $\omega_{*}$ from (8), we set

$$\begin{array}{c} \sqrt{\lambda }=m\omega_{*}=\frac{m\pi }{\int_{0}^{1}\sqrt{s(\tau )}\;d\tau }=\frac{m\pi }{2p+\sqrt{1+C}\left(1-2p\right)}\;, \end{array}$$

which yields

$$\begin{array}{c} \cos\left(u+v\right)=\cos\left(m\pi \right)=-1\;,\;\cos\left(u-v\right)=\cos\left(\frac{1}{2}m\pi \right)=0\;,\;m\in N_{odd}\;, \end{array}$$

so

$$\begin{array}{c} D\left(m\omega_{*};s_{\mathrm{step}}\right)=-\frac{1}{2}\left(2+\frac{2+C}{\sqrt{1+C}}\right)<-2\;,\;m\in N_{odd}\;, \end{array}$$

which is precisely what is stated in Lemma 1.    □

**Figure 3:**
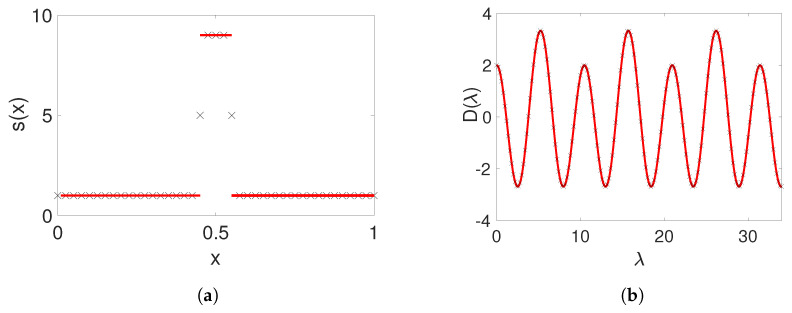
Comparison of exact computation and numerical approximation of the step-function heterogeneity and of the discriminant of Equation (9) (for p=0.45) using Matlab (ODE15s). (**a**) Exact step potential (7) *(solid red line)* and discretized version of the step function on the computational grid based on Equation (19) *(black crosses)*. (**b**) Exact discriminant (9) *(solid red line)* and numerical approximation of (5) using (19) *(black crosses).*

The upshot of this result is that the function s(x) and the breather frequency ω can be tuned to pairs $s_{\mathrm{step}},\omega_{*}$, resulting in a band structure with uniformly open, odd-numbered band gaps into which multiples of the breather frequency $\omega_{*}$ fall. This is the main driving force of the breather construction, namely designing the spectral properties of the linear part to avoid resonances, i.e., by avoiding these resonances, we engineer a frequency $\omega_{*}$ for which breather existence should not be precluded. Then, in our numerical explorations, we investigate both the frequency and other system parameters to showcase the existence and examine the stability of the relevant breathers in this continuum, heterogeneous medium. Note that the specific coefficients from [12] correspond to p=6/13 and have jump regions that are rather steep and thin—a feature that can be avoided by using a value of p<6/13 (as shown, e.g., in the right panel of Figure 2). For a comparison of the analytical and numerical setup of the function s(x) and the corresponding discriminant given in (9), see Figure 3.

**Figure 4:**
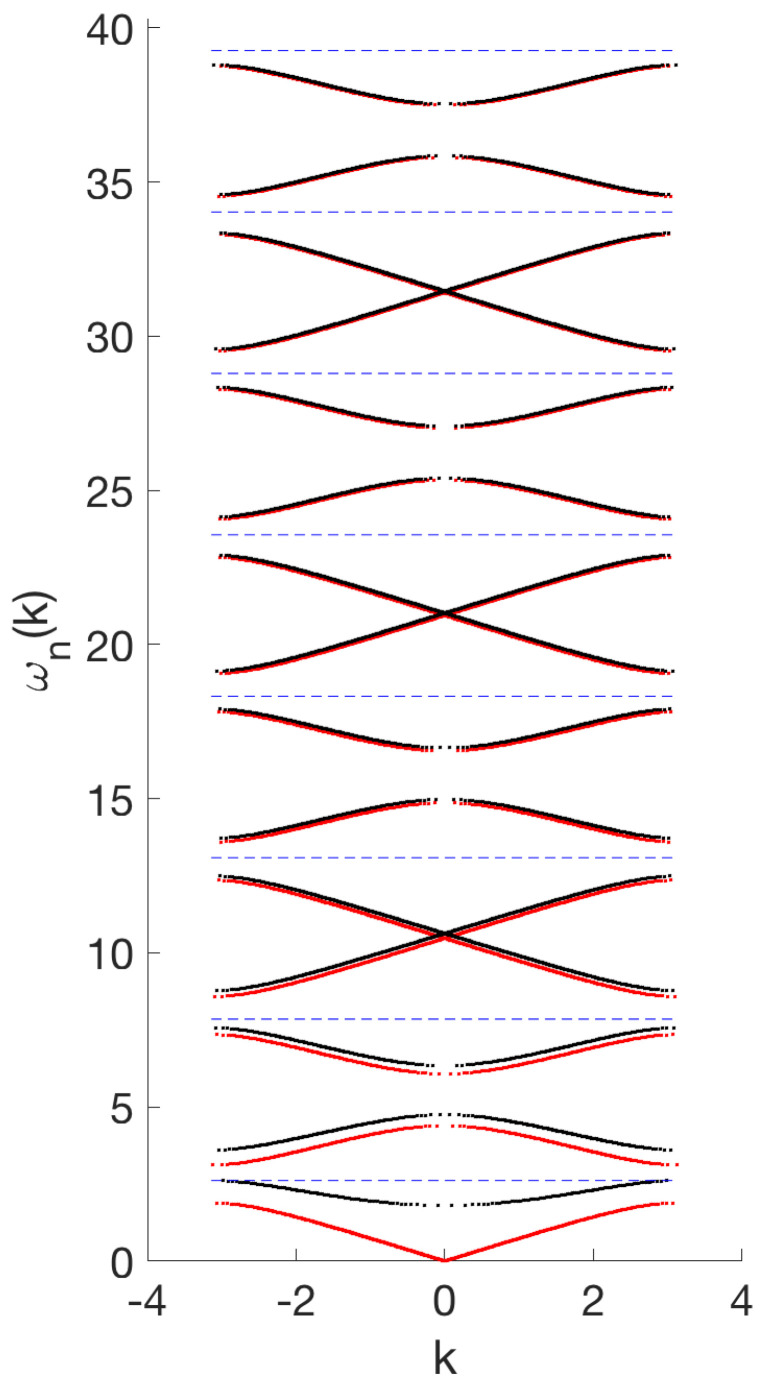
Exact band structure computed from the exact discriminant (9) (red) for $s=s_{\mathrm{step}},\;q=0$ vs. the numerically computed band structure for $s=s_{\mathrm{step}},\;q=q_{0}s_{\mathrm{step}}$ with a smoothed step function as in (19) using Matlab (black), along with odd multiples of $\omega_{*}$ from (8) (blue dashed lines). Parameter setting: p=0.45. Observe how the choice of $q_{0}$ puts the first band edge at $\omega_{*}$ and how the band structure eventually approaches the exact expression for q=0 for higher bands, just as predicted by theory. For $s=s_{\mathrm{step}},q=s\left(q_{0}-\varepsilon^{2}\right)$ with small ε, the frequency ($\omega_{*}$) moves slightly into a spectral gap.

So far, the coefficient *q* in (1) has been set to zero. Its role is to create a bifurcation of small-amplitude breathers from a band edge. The direct computation leading to (9) is difficult to carry out for non-zero *q*. We instead make use of a theoretical result according to which the band structure for the cases of q=0 and q≠0 (with the same *s* in both cases) only differs in the lower bands if $q\in L^{2}\left(0,1\right)$ (see, e.g., [20]). This can be illustrated numerically (see Figure 4 and Section 2.2 for an explanation of the numerical method).

**Definition** **2** (Resonance-free triplet)**.** *We call $\left(s_{\mathrm{step}},q_{\mathrm{step}},\omega_{*}\right)$ a “resonance-free triplet” if $s_{\mathrm{step}},\omega_{*}$ are as in Lemma 1 and $q_{step}\left(x\right):=s_{step}\left(x\right)\left(q_{0}-\varepsilon^{2}\right)$ for ε>0 with $q_{0}\in R$ chosen such that for the system,*

(10) $$\begin{array}{c} \frac{d}{dx}\Phi_{\omega_{*}}\left(x\right)=\left(\begin{array}{cc} 0 & 1\\ s_{step}\left(x\right)\left(q_{0}-\omega_{*}^{2}\right) & 0 \end{array}\right)\Phi_{\omega_{*}}\left(x\right)\;,\;\Phi_{\omega_{*}}\left(0\right)=Id\;, \end{array}$$

*the Floquet discriminant ($D(\omega_{*};s_{\mathrm{step}},q_{0},\varepsilon )$) fulfills the following:*

- *At ε=0, we have $D\left(\omega_{*};s_{\mathrm{step}},q_{0},0\right)=-2,D\left(m\omega_{*};s_{\mathrm{step}},q_{0},0\right)<-2$ for $1<m\in N_{\mathrm{odd}}$;*
- *For ε>0 sufficiently small, we have $D(\omega_{*};s_{\mathrm{step}},q_{0},\varepsilon )<-2$ for $m\in N_{\mathrm{odd}}$.*

The main idea behind introducing *q* in such a special way is to first determine $q_{0}$ (in terms of $s_{\mathrm{step}}$ and $\omega_{*}$) such that $\omega_{*}$ resides precisely on a band edge, then introduce a small bifurcation parameter ε>0 that pushes the first band edge down in such a way that $\omega_{*}$ slips into a spectral band gap. The breather is predicted to exist for ε>0 sufficiently small.

### 2.2. Existence Result and Its Numerical Implementation

**Proposition** **1** (Existence of breathers)**.** *Let ρ<0, and let $\left(s,q,\omega \right)=\left(s_{\mathrm{step}},q_{\mathrm{step}},\omega_{*}\right)$ be a resonance-free triplet as in Definition 2. Then, there is $\varepsilon_{0}>0$ such that for all $\varepsilon \in (0,\varepsilon_{0})$, there exists a breather solution of *(1)* that can be approximated by*

(11) $$\begin{array}{c} u_{\mathrm{breather}}\left(x,t\right)=\varepsilon \eta_{1}\;\mathrm{sech}\left(\eta_{2}\varepsilon x\right)q_{11}\left(x\right)\sin\left(\omega_{*}\left(t-t_{0}\right)\right)+h.o.t.\;,\;t_{0}\in R\;, \end{array}$$

*with*

(12) $$\begin{array}{cc} \eta_{1} & :=2\sqrt{\frac{2{\bar{s}}_{1}}{{\bar{s}}_{3}}}\;,\eta_{2}:=\sqrt{{\bar{s}}_{1}}\;,\\ {\bar{s}}_{1} & =-\left(\frac{1}{2\det S}\right)\int_{0}^{2}s_{\mathrm{step}}\left(x\right)q_{11}{\left(x\right)}^{2}\;dx\;,{\bar{s}}_{3}=\frac{3\rho }{2\det S}\int_{0}^{2}q_{11}{\left(x\right)}^{4}\;dx\;, \end{array}$$

*where $Q\left(x\right)={\left(q_{jl}\left(x\right)\right)}_{j,l=1,2}$ and S are defined as follows. Let $\Phi_{\omega_{*}}$ be a matrix solution for *(10)* and define Q(x):=P(x)S according to the following Floquet representation*

(13) $$\begin{array}{c} \Phi_{\omega_{*}}\left(x\right)=P\left(x\right)e^{xM}=P\left(x\right)Se^{xJ}S^{-1}\;,\;J=\left(\begin{array}{cc} 0 & 1\\ 0 & 0 \end{array}\right)\;,\;P\left(x+2\right)=P\left(x\right)\;. \end{array}$$

Proof sketch. In a first step, the time dependence is eliminated by representing *u* by its Fourier series in time, i.e.,

$$\begin{array}{c} u\left(x,t\right)=\sum_{n\in Z}{\hat{u}}_{n}\left(x\right)e^{in\omega t} \end{array}$$

which turns the PDE into a countably infinite nonlinear system, i.e.,

$$\begin{array}{c} 0={\hat{u}}_{n}^{\prime \prime }+\left(\omega^{2}s\left(x\right)+q\left(x\right)\right){\hat{u}}_{n}+g_{n}\left({\left({\hat{u}}_{k}\right)}_{k\in Z}\right)\;,\;n\in Z\;, \end{array}$$

for the (temporal) Fourier coefficients ${\hat{u}}_{n}$ and a convolution nonlinearity $g_{n}$. Upon restriction to an invariant subspace (arising through various symmetries that (1) is naturally equipped with), one is left with a similar countably infinite system of ODEs—but now for a real-valued variable $u_{n},n\in N_{\mathrm{odd}}$. The tuning of the band structure via the resonance-free triplet from Definition 2 results in a Floquet exponent configuration that allows for the use of a blend of center-manifold reduction for PDEs and bifurcation theory (see, e.g., [21]) to reduce the analysis to a planar ODE with periodic coefficients. This reduced ODE is treated via averaging methods and finally demonstrated to support homoclinic (to zero) orbits (explicitly expressed in terms of sech functions). These then finally yield the desired breather solution (see [12] for details).

**Remark** **1.** *It turned out to be computationally convenient in breather construction to take s,q to be 2- instead of 1-periodic, since in that case, the Floquet exponents turned out to be purely real. We do not further comment here on that (see [12]). Notice also that in our considerations we explicitly ensure that ${\bar{s}}_{3}\neq 0$ in the above Proposition.*

Related work. Note that breather solutions are closely related to standing and traveling modulated pulses/wave packets whose existence (on long but finite time scales) is usually demonstrated via derivation and justification of the nonlinear Schrödinger equation as an amplitude equation (see [22] for a single wave packet and [23] for wave-packet interaction). Furthermore, there are so-called generalized breather solutions that feature (small) periodic tails (see [24]) due to resonances with the band structure. The main motivation for studying nonlinear wave equations with spatially periodic coefficients comes from an application in photonics, where one strives to design periodic materials (represented by the periodic coefficients) such that there exist standing light pulses (represented by breather solutions) (see [25]).

Linear stability analysis. Linearizing around the breather solution (11) results in a linear operator, i.e.,

(14) $$\begin{array}{c} L_{\mathrm{breather}}\left(\begin{array}{c} u\\ v \end{array}\right):=\left(\frac{1}{s_{\mathrm{step}}\left(x\right)}\right)\left(\begin{array}{cc} 0 & 1\\ \partial_{x}^{2}+q_{\mathrm{step}}\left(x\right)+3\rho u_{\mathrm{breather}}{\left(x,t\right)}^{2} & 0 \end{array}\right)\left(\begin{array}{c} u\\ v \end{array}\right)\;. \end{array}$$

The time periodicity of the breather results in the use of Floquet theory in time. Due to the temporal translation invariance of the PDE, one immediately finds that $\left[\partial_{t}u_{\mathrm{breather}}\left(x,0\right),\partial_{t}^{2}u_{\mathrm{breather}}\left(x,0\right)\right]$ is an eigenfunction belonging to the Floquet multiplier +1 (or Floquet exponent 0). This eigenfunction is associated with the so-called phase mode. Furthermore, the essential spectrum is related to the band structure of the linear PDE (2). Analytically retrieving any further information on the spectrum is difficult. Hence, in the remaining part of the manuscript, we turn to numerical methods to analyze the stability of the constructed breather structures.

Numerical computation of $q_{0}$ from Definition 2. After choosing p∈(3/8,1/2), one can compute $s_{\mathrm{step}}$ and, therefore, $\omega_{*}$, as in Lemma 1. This then allows for the computation of $q_{0}$ by numerically defining a function $T\left(\mu \right):=\mathrm{trace}\left(\Phi_{\omega_{*}}\left(x;s_{\mathrm{step}},\mu ,0\right){|}_{x=2}\right)+2\;$, with $\Phi_{\omega_{*}}$ being the canonical fundamental matrix corresponding to (10). We suppress its dependence on $\left(s_{\mathrm{step}},\omega_{*}\right)$, since these are kept fixed, while we vary $q_{0}$ in $q_{\mathrm{step}}\left(x\right)=s_{\mathrm{step}}\left(x\right)q_{0}$. Numerical root finding for $T(q_{0})=0$ yields $q_{0}$ (upon having a good enough first guess). Note that there is also a way of finding an explicit formula for $q_{0}$ (see [12]) in the step-function case, but this is of limited numerical use, since it turns out to be more convenient to perform numerical computations for smoothed step functions.

Numerical band-structure computation for the linear problem. Let us now also briefly discuss how to numerically implement the computation of the band structure for the linear PDE (2). Using Floquet theory, one can compute for which values of ω there exists a bounded solution *v* of (3) by inspecting the Floquet exponents (which determine if a fundamental system for (3) yields bounded solutions or exponential growth/decay). In particular, there is a relation between the Floquet exponents ${\tilde{l}}_{\pm }={\tilde{l}}_{\pm }\left(\omega \right)\in C$ and the spectral parameter ω expressed by

(15) $$\begin{array}{c} e^{{\tilde{l}}_{\pm }}=\frac{1}{2}\left(D\left(\omega \right)\pm \sqrt{D{\left(\omega \right)}^{2}-4}\right)\;. \end{array}$$

Moreover, for our equation, the Floquet exponents are either purely real or purely imaginary (with the usual non-uniqueness from possibly adding multiples of 2πi due to the periodicity of the complex exponential). One can now choose values of ω∈R and numerically compute the following for each such ω: (1) the canonical fundamental matrix $\Phi_{\omega }$ for (6); (2) the Floquet exponents from (15); and, finally, (3) if |D(ω)|<2, then ${\tilde{l}}_{\pm }=\pm il\in iR$ and ω belongs to a spectral band. Plotting the relation of ±l vs. ω (for ω in a spectral band) yields the band structure, while simply plotting ω belonging to a spectral band yields the “banded” spectrum (see Figure 4 for a numerical computation of the band structure for $q\left(x\right)=q_{0}s_{\mathrm{step}}\left(x\right)$).

Numerical computation of the breather approximation $u_{\mathrm{breather}}$ from (11). Equipped with a resonance-free triplet $\left(s_{\mathrm{step}},q_{\mathrm{step}},\omega_{*}\right)$, one can numerically compute the canonical fundamental matrix $\Phi_{\omega_{*}}\left(x;s_{\mathrm{step}},q_{0},0\right)$. Using Floquet theory, one can derive the representation of (13), which, in particular, yields $\Phi_{\omega_{*}}\left(2\right)=e^{2M}$ such that *M* can be determined by numerically computing the matrix logarithm. Note that a Jordan block is expected to arise (see [12] for details), so computing *S* and *J* numerically can be a delicate task. Finally, setting $P\left(x\right)=\Phi_{\omega_{*}}\left(x\right)e^{-xM}$ and Q(x)=P(x)S enables the computation of all necessary constants in (12) by numerical integration. See Figure 1 for an illustration of such a computation.

## 3. Stability of Breathers

We now numerically consider the abovementioned setup of a resonance-free triplet (2) towards identifying a numerically exact (up to a prescribed accuracy) breather waveform. Throughout this section, we fix p=0.45 and ε=0.3. This value of ε is selected so that the breather is localized for the chosen values of the domain length and *p*. More concretely, the breather’s FWHM in this case is smaller than one-eighth of the domain length. We checked that the results below are similar for p=0.44 and ε∈[0.15,0.45].

In order to practically identify breather solutions, we discretize the relevant Klein–Gordon PDE. Among all the discretization schemes, we chose to utilize finite differences, for simplicity but also confirmed their ability to capture theoretically predicted features, as discussed below. To this aim, we take a uniform grid whose lattice spacing is given by *h*. Due to the large domain needed to contain the breather, we need to select a value of *h* that yields a tractable lattice size. The domain extends over the interval of [−L,L) (with periodic boundary conditions), and the number of lattice sites is N=2L/h (for simplicity, *N* is taken as an even integer number). In our numerics, we take h=0.02 and L=60 so that N=6000. The value of *x* at the lattice nodes, i.e., $x_{n}\equiv x\left(n\right)$, is dependent on the choice of *p*. For the numerics, we take *p* with two decimal digits, and have chosen $x_{n}=nh$ if 100p is even and $x_{n}=\left(n+1/2\right)h$ if 100p is odd.

With this discretization, we can write (1) as

(16) $$s_{n}{\ddot{u}}_{n}=\frac{1}{h^{2}}\Delta u_{n}-q_{n}u_{n}-u_{n}^{3}\;,\;n=-N/2\ldots N/2-1$$

with $u_{n}\equiv u\left(x_{n}\right)$ and $q_{n}=s_{n}\left(q_{0}-\varepsilon^{2}\right)$. In order to obtain a good correlation between the analytical and numerical spectrum for linear modes, a sixth-order discretization for $\partial_{x}^{2}$ is introduced [26]. That is,

(17) $$\Delta u_{n}=\frac{1}{90}\left(u_{n-3}+u_{n+3}\right)-\frac{3}{20}\left(u_{n-2}+u_{n+2}\right)+\frac{3}{2}\left(u_{n-1}+u_{n+1}\right)-\frac{49}{18}u_{n}.$$

Numerically, from a practical perspective, we find the choice of $s_{n}$ to be central towards the convergence of our numerical scheme. Here, we define the function of s(x) so that just in the step (i.e., for x=p or x=1−p), the value of s(x) takes an intermediate value, i.e., 1+C/2. In other words, $s_{n}$ is taken as

(18) $$s_{n}=1+C\times \left\{\begin{array}{cc} 0, & x_{n}<p\\ 1/2, & x_{n}=p\\ 1, & p<x_{n}<1-p\\ 1/2, & x_{n}=1-p\\ 0, & x_{n}>1-p \end{array}\right.\;\mathrm{if}\;x_{n}\in \left[0,1\right).$$

**Figure 5:**
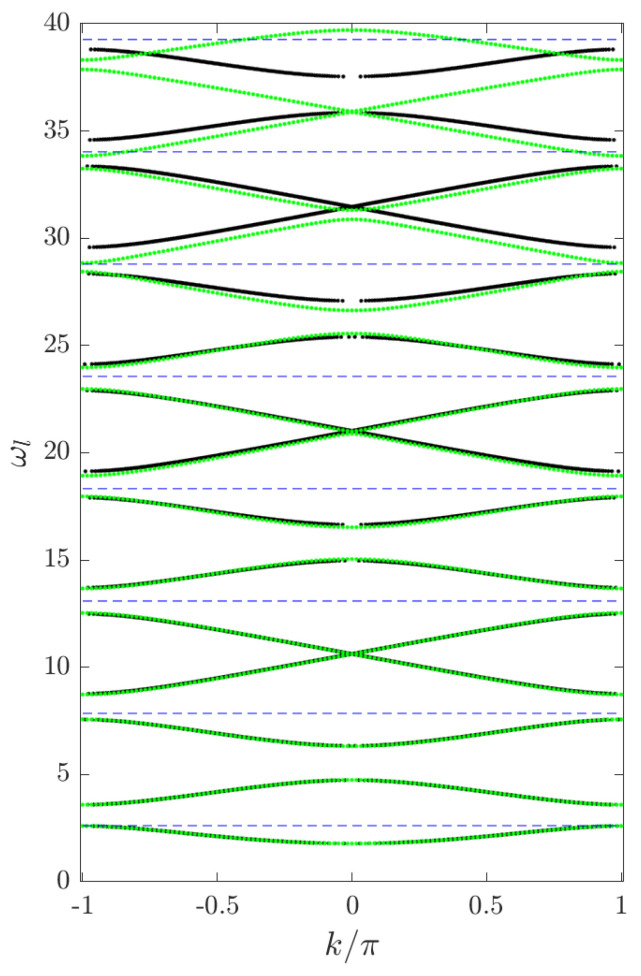
Numerical linear dispersion for p=0.45 obtained from (20) (green dots) and numerically computed dispersion relation via Floquet discriminant (black dots) for s(x) as in (19). The frequency ($\omega^{*}$) and its odd multiples up to 15 are indicated by blue dashed lines.

For our numerical purposes, the choice of s(x) that we find to correlate efficiently with this requirement is an approximated step function in the following form:

(19) $$s\left(x\right)=1+\frac{C}{2}\left[\tanh(\mu (x-p))+\tanh(-\mu (x-(1-p)))\right],$$

with a high value of μ, such as $10^{5}$. This choice yields a value of $\omega^{*}=2.61778$, which is very close to the analytical value corresponding to the step function, namely $\omega^{*}=3\pi /\left(8p\right)=2.61800$. This choice also yields a value $q_{0}=3.2701$ for the constant appearing in Definition 2. We compared the exact band structure determined in Section 2.2, stemming from the linearization of (1), with the structure arising from the linearization of (16). In other words, if one introduces the linear mode expression $u_{n}\left(t\right)=U_{n}\exp\left(i\omega_{l}t\right)$ at (16) around the trivial equilibrium ($u_{n}=0$), the following generalized eigenvalue problem is obtained:

(20) $$\omega_{l}^{2}s_{n}U_{n}=\left(\frac{1}{h^{2}}\Delta -q_{n}\right)U_{n},$$

whose numerical diagonalization yields the spectrum of linear modes $\omega_{l}$. Figure 5 shows the analytical and numerical linear mode spectra for p=0.45 and the odd-integer multiples of $\omega^{*}$. One can see that there are resonances with the numerical spectrum for the 13th (and higher) harmonic. However, as we explain below, they do not impact the existence of breathers.

In order to obtain time-reversible breathers from (16), one can work in the Fourier space by expanding $u_{n}\left(t\right)$ into associated modes according to the following expression:

(21) $$u_{n}\left(t\right)=z_{0,n}+2\sum_{k=1}^{K}z_{k,n}\cos\left(k\omega t\right),\;z_{k,n}\equiv z_{k}\left(x_{n}\right)$$

Thus, (16) transforms into a set of (K+1)N nonlinear algebraic equations ($F(\left\{z_{k,n}\right\})=0$) as follows:

(22) $$F_{k,n}\equiv -k^{2}\omega^{2}s_{n}z_{k,n}-\frac{1}{h^{2}}\left(z_{k,n+1}+z_{k,n-1}-2z_{k,n}\right)+q_{n}z_{k,n}+F_{k,n}=0.$$

where $F_{k,n}$ denotes the *k*-th mode at the *n*-th site of the discrete cosine Fourier transform of $u_{n}^{3}$, i.e.,

(23) $$F_{k,n}=\frac{1}{2K+1}\left[u_{n}^{3}\left(0\right)+2\sum_{q=1}^{K}u_{n}^{3}\left(t_{q}\right)\cos\left(k\omega t_{q}\right)\right],$$

with $u_{n}\left(t\right)$ taken from (21) and $t_{q}=2\pi q/\left(\left(2K+1\right)\omega \right)$.

**Figure 6:**
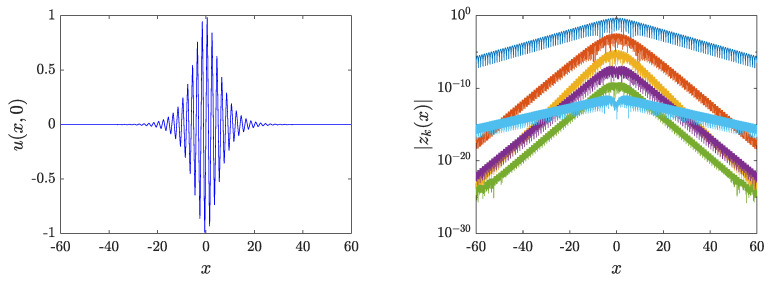
Breather at p=0.45 and $\omega =\omega^{*}$. The left panel shows the profile at t=0, while the right panel shows the odd-*k* Fourier coefficients ($|z_{k}\left(x\right)|$) on a semilogarithmic scale.

**Figure 7:**
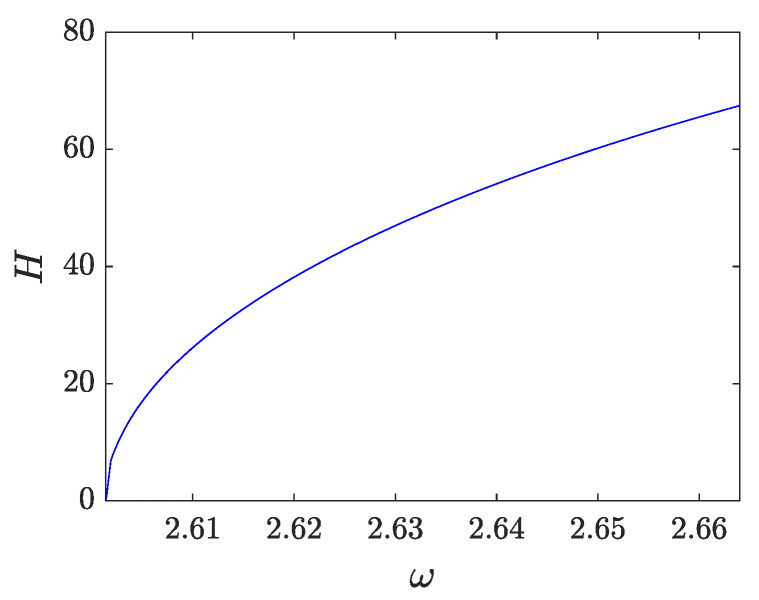
Dependence of energy as a function of the breather frequency for p=0.45.

To solve (22), we make use of fixed-point methods. Among those methods, we choose the trust-region dogleg approach, which is the default algorithm in Matlab’s fsolve function. In order to implement the fixed-point method, we set the initial guess as $z_{k\neq 1,n}=0,\;z_{1,n}=u_{\mathrm{breather}}\left(x_{n},0\right)$ where $u_{\mathrm{breather}}$ refers to the analytical breather approximation of Equation (11). Figure 6 shows the profile of the breather for $\omega =\omega^{*}$ and p=0.45. This breather can be continued until a resonance with linear modes occurs. Taking into account that the breather possesses harmonics up to the *K*-th mode (within our Ansatz), only resonances of $k\omega^{*}$ with odd k≤K are considered. With those constraints and accounting that we have set K=11, the resonances with the 13th (and higher) harmonic observed in Figure 5 are irrelevant for our considerations herein. For the choice of p=0.45, there are no resonances when choosing ω∈(2.6014,2.6221). The lower bound within the interval corresponds to the first harmonic resonance with the top of the first band, whereas the upper bound holds for the resonance of the eleventh harmonic with the bottom of the tenth band. However, the breather can be continued past the latter boundary, as the effect of such resonance is introducing a modification of the breather of the order of $10^{-8}$. Similarly, the next resonance, which occurs for ω=2.6639 and comes from the ninth harmonic, also has a negligible effect (of the order of $10^{-7}$). Figure 7 shows the energy versus ω for p=0.45, where one can see that the energy tends to zero at the lower bound of the interval (as the breather bifurcates from the corresponding π phonon at the upper edge of the band). The energy of the breather is equivalent to the Hamiltonian associated with (1), i.e.,

(24) $$H=\int \left[\frac{1}{2}s\left(x\right){\left(\partial_{t}u\left(x,t\right)\right)}^{2}+\frac{1}{2}{\left(\partial_{x}u\left(x,t\right)\right)}^{2}+\frac{1}{2}q\left(x\right)u^{2}\left(x,t\right)+\frac{1}{4}u^{4}\left(x,t\right)\right]\;dx$$

The stability properties of the obtained solutions are identified by means of Floquet analysis. To that effect, we add a perturbation ξ(x,t) to the solution u(x,t) of (1). The resulting linearized PDE reads as follows:

(25) $$s\left(x\right)\partial_{t}^{2}\xi =\partial_{x}^{2}\xi -\left(q\left(x\right)+u^{2}\right)\xi$$

The aim of the Floquet analysis is to compute the spectrum of the Floquet operator, whose matrix representation is known as the monodromy matrix (M), which is defined according to the following map:

(26) $$\Omega \left(T\right)=M\Omega \left(0\right),\;\Omega \left(t\right)=[\xi \left(x,t\right),\partial_{t}\xi \left(x,t\right)]$$

The eigenvalues of M represent the Floquet multipliers and can be written as λ=exp(iθ). Given the real, symplectic nature of the Floquet operator, the multipliers come in pairs (λ,1/λ) if they are real or in quadruplets $\left(\lambda ,\lambda^{*},1/\lambda ,1/\lambda^{*}\right)$ if they are complex. For a periodic solution to be stable, generally, |λ|≤1 must hold. In our more specific Hamiltonian setting, stability necessitates that all the eigenvalues lie on the unit circle.

**Figure 8:**
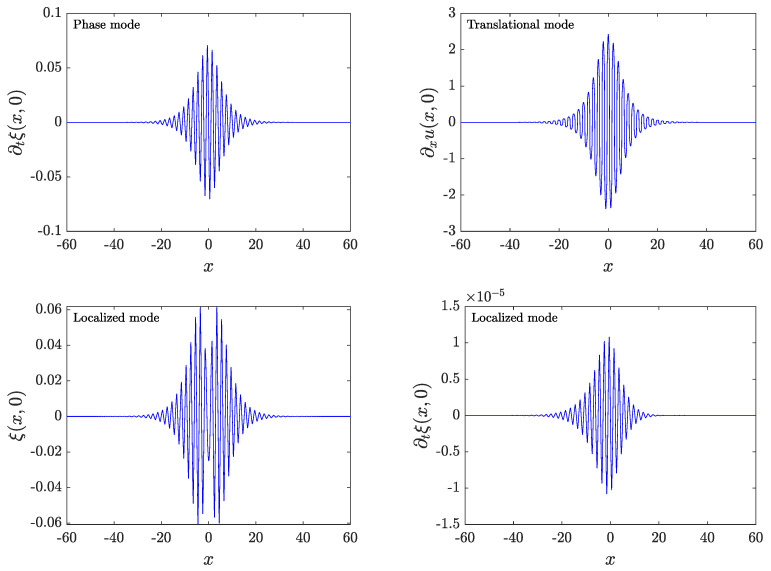
(**Top left**) Non-zero component of the phase mode. (**Top right**) Translational mode defined as $\partial_{x}u\left(x,0\right)$. (**Bottom**) Components of the localized mode. In every panel, p=0.45 and $\omega =\omega^{*}$.

More precisely, due to the invariance of our considered model under time translation, there is an eigenvalue pair at 1+0i. As mentioned after (14), its associated eigenmode, known as the phase mode, corresponds with $\Omega \left(t\right)=[\partial_{t}u\left(x,t\right),\partial_{t}^{2}u\left(x,t\right)]$. In order to numerically find the monodromy spectrum, (25) must be discretized on the same grid as that where the solution was identified. Then, we perform simulations of these linearization equations for a period. To this end, we use the fourth-order explicit and symplectic Runge–Kutta–Nyström method developed in [27] with a time step of δt=T/1500 and T=2π/ω. With this choice, for a breather with a frequency $\omega^{*}$ and p=0.45, we find that the mode associated with the above symmetry is at $\theta \approx 9\times 10^{-7}$; this serves as a benchmark for the accuracy of our Floquet multiplier computations. Very close to 1+0i, we can find a real localized mode at a distance of $\approx 5\times 10^{-7}$ from 1+0i, indicating an instability (although with an extremely small growth rate). Figure 8 shows the shapes of both modes. Notice that, on the one hand, for the phase mode, ξ(x,0)=0 because of the time-reversibility of the breather; on the other hand, one might think that the localized mode could be related to a translational mode ($\xi \left(x,0\right)=\partial_{x}u\left(x,0\right)$), a feature that can occur in discrete Klein–Gordon lattices [28]. However, as shown in Figure 8, this localized mode has a different shape than the translational mode. In addition, the $\partial_{t}\xi \left(x,0\right)$ component of the localized mode is not zero. Although in the case shown in Figure 8, it is tiny compared to the ξ(x,0) component, it grows with ω, and, for instance, it is only eight times smaller when ω=2.66. This suggests that some velocity may be imparted on the structure upon perturbation and may accordingly lead to potential mobility (the relevant dynamics are discussed below).

**Figure 9:**
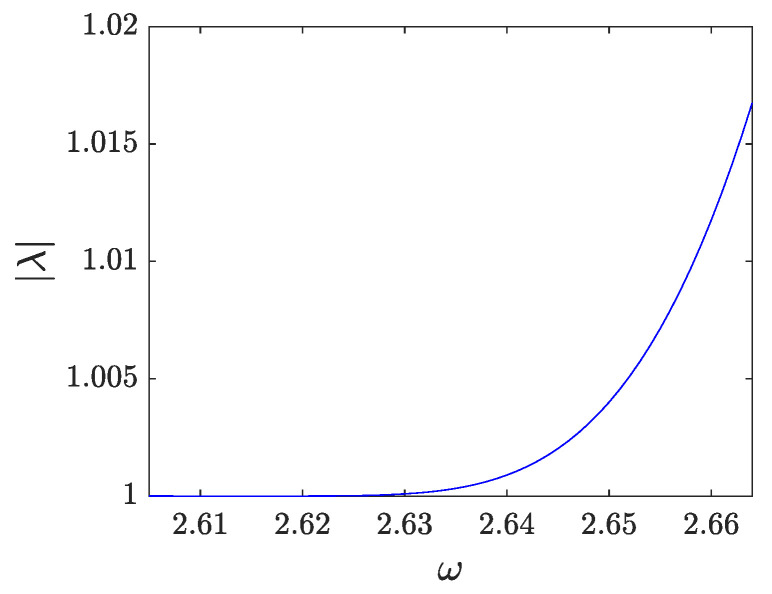
Dependence of the modulus of the multiplier associated with the localized mode with respect to the frequency for p=0.45.

When the frequency is varied from $\omega^{*}$, the localized mode is always real and higher than 1 and exhibits monotonically increasing behavior with ω. As |λ|≥1 in all the considered intervals, the breather is unstable.The dependence of the corresponding instability growth rate, as reflected in the real Floquet multiplier pertaining to the instability is shown in Figure 9.

**Figure 10:**
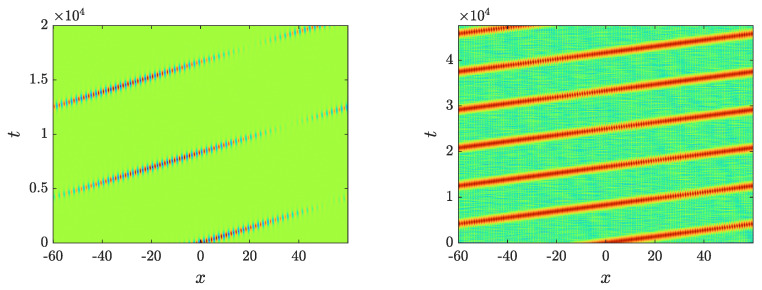
Evolution of the breather wavefunction (u(x,t)) (**left**) and the logarithm (base 10) of the energy density (h(x,t)) (**right**) with respect to time (**left**) for a moving breather with ω=2.64 and p=0.45, generated by a perturbation of δ=0.05.

In order to explore the effect of the instability caused by the localized mode, we simulated (1) with the perturbed stationary breather as initial condition, which, taken in the form of $u\left(x,0\right)=\tilde{u}\left(x,0\right)$, $\partial_{t}u\left(x,0\right)=\delta \;\xi \left(x,0\right)$, with $\tilde{u}\left(x,t\right)$ being the breather solution to (1) and δ being the perturbation strength, while ξ(x,0) is the corresponding component of the localized eigenmode. Interestingly, the perturbed breather starts moving with a constant speed, as can be observed in Figure 10, which shows the evolution of the moving breather with ω=2.64 for a perturbation δ=0.05. This figure displays both the wavefunction profile u(x,t) for short times and the energy density h(x,t) for longer times; the latter arises from the definition of the Hamiltonian (24) so that H=∫h(x)dx. For this and other simulations, a time step of δt=T/100≈0.026 was chosen, which conserves the energy with a relative error ~$10^{-8}$.

**Figure 11:**
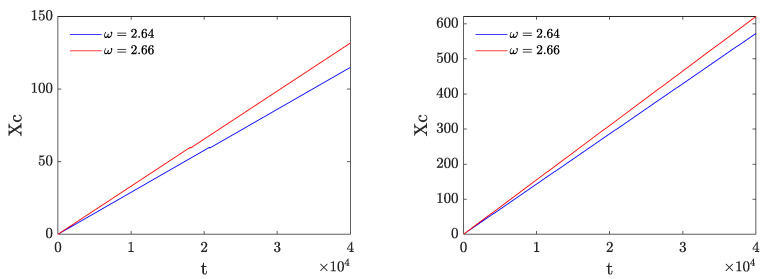
Evolution of the energy center for moving breathers with ω=2.64 and ω=2.66 obtained by adding a perturbation with amplitudes of δ=0.01 (**left**) and δ=0.05 (**right**).

A consequence of the smoothness of the motion can be observed in Figure 11, where the time dependence of the energy center, defined as

(27) $$X_{c}=\frac{1}{H}\int xh\left(x\right)\;dx,$$

is plotted for breathers with ω=2.64 and ω=2.66 perturbed by δ=0.01 and δ=0.05. Remarkably, the relevant energy center follows a straight line, which, in turn, clearly indicates that the breather moves with constant velocity despite the emitted radiation.

These results seem to strongly point to the instability of our stationary breather state towards moving breathers, although the localized eigenmode is different than the translational mode. One explanation of this discrepancy relies on the fact that the projection of the localized mode onto a translational-type mode (i.e., one that leads to mobility) is large enough so that the perturbation is able to lead to a breather motion. They also clearly seem to point towards the existence of exact traveling breather waveforms, which would be of particular interest to identify in the so-called co-traveling frame (traveling with the breather). Nevertheless, this is a substantial task in its own right that is deferred to future publications.

We close this section by noting the intriguing feature of the periodic vanishing of the field observed in Figure 10. Probing the dynamics, we find that the vanishing frequency is ~$8.7\times 10^{-4}$. This phenomenon, which might have its origin in the fact that the unstable eigenmode is not a purely translational mode, appears over a time scale of the order of the inverse of the rate of growth of the unstable mode. For this particular breather, the growth mode is ~$3.7\times 10^{-4}$, so its inverse is roughly within the same ballpark as the observations of Figure 10. Of course, once the nonlinear dynamics of the evolution of the instability set in, the dynamics are less predictable, yet the relevant field appears to vanish in the left panel of the figure.

## 4. Conclusions and Future Challenges

In the present work, we revisited the important and interesting topic of the potential existence of breather-type waveforms in continuous, nonlinear media. The current pervasive impression in the nonlinear community is that such waveforms are absent in generic nonlinear wave equations, except for the setting of integrable models such as the sine-Gordon equation (or, similarly, the modified Korteweg–de Vries equation). Nevertheless, recent significant mathematical developments have paved the way towards the identification of such exact (up to a prescribed accuracy) waveforms in continuum, nonlinear media, most notably for PDEs of the Klein–Gordon type bearing (suitably designed) spatial heterogeneity.

While these efforts have provided a theoretical backdrop for the existence of such breather waveforms, to the best of our knowledge, such states have not previously been systematically computed, nor has their stability been elucidated. This is a primary contribution of this work, where such solutions are identified via a Fourier space method and, subsequently, their Floquet analysis is explored, as parameters such as the frequency ω of the breather, as well as those of the model (such as *p*) are varied. We were generally able to identify the theoretically established waveforms. We have also shown that the considered examples all feature a real pair of Floquet multipliers associated with the spectral instability of the relevant breathers. This instability was dynamically explored in its own right, showcasing the potential of these breathers to travel when perturbed in the pertinent unstable eigendirections of the standing breather state.

Naturally, this work paves the way for numerous additional questions that can be addressed in future studies. One of these certainly concerns the direct computation (as exact solutions in a co-traveling frame) of the traveling waveforms, as well as the potential examination of their stability. On the other hand, the instability of stationary breathers investigated herein also poses the question of whether there can be other variants of this (or other similar) class of models where heterogeneous, continuum, stationary breathers can actually be dynamically robust and spectrally stable. At a larger scale, given the theoretical understanding of the requirements for establishing such structures, it is also relevant to consider optimization problems enabling the design of “optimal nonlinear media” towards, e.g., the widest possible interval of existence of breather waveforms. Some of these topics are presently under consideration, and relevant findings will be reported in future publications.

## Data Availability

The data pertaining to this study are available upon reasonable request from the authors.
